# Application of Antiviral, Antioxidant and Antibacterial *Glycyrrhiza glabra* L., *Trifolium pratense* L. Extracts and *Myristica fragrans* Houtt. Essential Oil in Microcapsules

**DOI:** 10.3390/pharmaceutics15020464

**Published:** 2023-01-30

**Authors:** Jurga Andreja Kazlauskaite, Inga Matulyte, Mindaugas Marksa, Raimundas Lelesius, Alvydas Pavilonis, Jurga Bernatoniene

**Affiliations:** 1 Department of Drug Technology and Social Pharmacy, Medical Academy, Lithuanian University of Health Sciences, LT-50161 Kaunas, Lithuania; 2 Institute of Pharmaceutical Technologies, Medical Academy, Lithuanian University of Health Sciences, LT-50161 Kaunas, Lithuania; 3 Department of Analytical and Toxicological Chemistry, Medical Academy, Lithuanian University of Health Sciences, LT-50161 Kaunas, Lithuania; 4 Institute of Microbiology and Virology, Veterinary Academy, Lithuanian University of Health Sciences, LT-47181 Kaunas, Lithuania

**Keywords:** plant extract, essential oil, microcapsules, extrusion, antioxidant activity, antiviral activity, antimicrobial activity, *Glycyrrhiza glabra* L., *Trifolium pratense* L., *Myristica fragrans* Houtt. essential oil

## Abstract

Viruses and bacteria can disrupt normal human functions; therefore, ways to use the beneficial properties of plants to promote health are constantly being researched. Plant materials that accumulate biologically active compounds can be used to create a new pharmaceutical form. This study aimed to investigate the biological activity of selected plant extracts and essential oil and to produce microcapsules. The main compounds in extracts and essential oil were determined using chromatographic methods, antioxidant activity was evaluated spectrophotometrically, antimicrobial activity was assessed by monitoring the growth of nine pathogens, and the antiviral effect on infected bird cells with coronavirus was evaluated. *Trifolium pratense* L. extract had the highest antioxidant (26.27 ± 0.31 and 638.55 ± 9.14 µg TE/g dw by the DPPH and ABTS methods, respectively) and antiviral activity (56 times decreased titre of virus). Liquorice extract expressed antibacterial activity against Gram-positive pathogens and the highest antioxidant activity using the FRAP method (675.71 ± 4.61 mg FS/g dw). Emulsion stability depended on excipients and their amount. Microcapsules with extracts and essential oil were 1.87 mm in diameter, and their diameter after swelling was increased more than two times in intestinal media, while less than 0.5 times in gastric media.

## 1. Introduction

Plant extracts and essential oils are used in many domains, including medicine, nutrition, flavouring, beverages, dyeing, repellents, fragrances, pharmaceutics, and cosmetics. Many plants have been recognized to have medicinal properties and beneficial impacts on health, e.g., antioxidant activity, digestive stimulation action, anti-inflammatory, antimicrobial, hypolipidemic, antimutagenic effects, and anticarcinogenic potential [1].

Phenolic compounds are one of the plants’ most evident secondary metabolites, and their distribution is demonstrated throughout the entire metabolic process. The biological activity of plants is primarily attributed to these compounds. Polyphenols contain numerous varieties of compounds: simple flavonoids, phenolic acids, complex flavonoids, and coloured anthocyanins [2,3,4].

Botanical substances obtained from plants, such as *Trifolium pratense* L. flowers, *Glycyrrhiza glabra* L. roots, and *Myristica fragrans* Houtt. essential oil, which are generally perceived as safe because of their natural origin, can be used for the prevention or treatment of menopausal health issues, various degenerative disorders, and possess many pharmacological activities, such as antiviral, antimicrobial, anti-inflammatory, antitumor, and other activities [5,6,7,8,9,10,11]. These plant substances could act synergistically and increase positive effects.

*Trifolium pratense* L. flower extracts are rich in isoflavones that possess estrogenic activity. However, it also has antioxidant and antibacterial activities, which may result from the presence of other flavonoids and phenolic compounds such as phenolic acids, clovamides, and saponins [12]. It is possible to obtain a preparation with strong antibacterial and antioxidant properties using *Trifolium pratense* L. extract with other plants.

Glycyrrhizin, a triterpenoid saponin glycoside, is considered the main bioactive component (4–10%) of *Glycyrrhiza glabra* L. roots (*Glycyrrhiza glabra* L.). It has been shown to possess glucocorticoid-like pharmacological effects and anti-inflammatory, antiviral, anti-tumour, and hepatoprotective activities [13]. A liquorice biological effect could be used in drugs and food supplements formulation, also it could be used in cosmetic products or in the food industry as a food and flavour additive [14]. Like *Trifolium pratense* L., the biological properties of this plant are due to phenolic compounds. Nevertheless, biological properties in plants can be provided not only by phenolic compounds in extracts but also by active compounds found in essential oils.

Essential oils contain important classes of compounds such as monoterpenes (C10 hydrocarbons based on two isoprene units), phenylpropanoids (C6 aromatic compounds with C3 side chains), sesquiterpenes (C15 hydrocarbons based on three isoprene units), diterpenes (C20), triterpenes (C30) and their oxygenated derivatives, and phenolic compounds (such as thymol and carvacrol) [15]. Several scientific researchers report that *Myristica fragrans* Houtt. essential oil has potential antioxidant, antimicrobial, anti-inflammatory, antiulcer, anticancer, aphrodisiac, and other activities caused by monoterpenes and phenolic compounds [10,16,17]. Not only can essential oils be used because of their biological properties but also because of the pleasant smell they can provide for the final product [18].

In order to protect volatile compounds found in essential oil to produce the final pharmaceutical, microencapsulation can be applied. Microencapsulation is a method that protects unstable compounds from environmental exposure. The excipients, which formulate a shell, could protect core substances.

Resistance to antibiotics has been on the rise in recent years. This is largely due to bacteria maintaining a genetic capacity to transmit and gain resistance against therapeutic compounds. In addition, many new multiresistant strains have arisen, which poses a serious threat both to individuals who are immunosuppressed as well as those who receive general hospital care [19,20]. Antibacterial agents derived from plants are a promising option for use in the treatment of infections. Plants lack some of the negative secondary effects and are often available and affordable.

Plant extracts that possess antiviral activity can be a valuable addition to an antiviral therapy. Antiviral drugs should target exactly the virus, but since viral particles live inside the host and gets integrated into the host cell, application of antiviral therapy through drugs is very difficult. Antiviral drugs may also kill the host cell [21]. Therefore, by using plant extracts that possess antiviral activity, the effective additional form or nutraceutical-like microcapsules can be created.

Sodium alginate is one of the most popular excipients used in extrusion [22,23,24,25,26]. It is a chemically stable polysaccharide and can form strong gel barriers from water [27,28]. Extrusion is a simple method that does not require expensive devices; for example, a medical syringe, syringe pump, and a crosslinker solution could be used to form particles [29,30]. Microcapsules prepared by extrusion could be used in wet and dry forms depending on the properties that are needed. This method’s particle size usually ranges from 0.25 to 2.5 mm [31,32]. The size depends on the shell material, crosslinker solution and concentration, and the amount of excipient(s) [33].

Microcapsules could be used to create and supplement products for pharmacy, cosmetics, food, and other industries. Moreover, microcapsules increase the shelf-life of labile compounds [34,35]. This form was selected because it could be used as a final product or incorporated into many pharmaceutical forms in order to obtain different release effects. Using plant extracts and essential oils made by microencapsulation is an excellent opportunity to conduct research, evaluate the properties of active compounds, and use them in developing new products.

In this research, three plants were chosen: *Trifolium pratense* L. flower, *Glycyrrhiza glabra* L. roots, and *Myristica fragrans* Houtt seeds. Their extracts and essential oil were used to formulate an emulsion and microcapsules by extrusion, and then their physical properties were evaluated. The main active compounds were determined and antioxidant, antimicrobial, and antiviral effects were assessed from *Trifolium pratense* L. and *Glycyrrhiza glabra* L. extracts and *Myristica fragrans* Houtt. essential oil.

This research could help create microcapsules with high antibacterial and antiviral effects and could be incorporated into a pharmaceutical form.

## 2. Materials and Methods

### 2.1. Plant Material and Reagents

*Trifolium pratense* L. samples were collected in *Trifolium pratense* L. fields in Laičiai, Kupiškis district, Lithuania (latitude 55°53024.2″ N; longitude 25°19036.0″ E). The collections of *Trifolium pratense* L. flower buds were made on the 31 July 2021. *Glycyrrhiza glabra* L. roots (the country of origin is China) were bought from LSMU pharmacy (Kaunas, Lithuania). Myristica fragrans seeds’ country of origin was Grenada (supplier Spaisvilė, Pašaltuonys, Lithuania). Voucher specimens (*Trifolium pratense* L.—J21731; *Myristica fragrans* Houtt.—I18922; and Glycyrrhiza glabra L.—K20911) were placed for storage at the Herbarium of the Department of Drug Technology and Social Pharmacy, Lithuanian University of Health Sciences, Lithuania.

In this experiment, purified water was prepared with GFL2004 (GFL, Burgwedelis, Germany). Deionised water was prepared with Millipore, SimPak 1 (Merck, Darmstadt, Germany). The following reagents were used: standards genistein, daidzein, and glycyrrhizin acid (Sigma Aldrich, Steinheim, Germany). 2,2′-azino-bis(3-ethylbenzothiazoline-6-sulfonic acid) (ABTS), 2,2-diphenyl-1-picrylhydrazyl radical (DPPH), and *β*-CDs purchased from Sigma Aldrich (Hamburg, Germany); aluminium chloride, hexaethylenetetraamine, dimethyl sulfoxide (DMSO), acetic acid, and Sabouraud dextrose agar (dehydrated) obtained from Sigma-Aldrich (Buchs, Switzerland); potassium persulfate obtained from Alfa Aesar (Karlsruhe, Germany); ethanol (96%) obtained from Vilniaus Degtinė (Vilniaus, Lithuania); Folin–Ciocalteu’s phenol reagent (Merck, Darmstadt, Germany); monosodium phosphate, ferrous sulfate heptahydrate, saline phosphate buffer, and hydrogen peroxide obtained from Sigma Aldrich (Schnelldorf, Germany); disodium hydrogen phosphate obtained from Merck (Darmstadt, Germany); Mueller–Hinton Agar obtained from BBL (Baltimore, MD, USA); foetal bovine serum obtained from FBS (Gibco, TX, USA); and as the shell material, alginic acid sodium salt from brown algae obtained from Sigma-Aldrich (Shanghai, China) was used. Calcium chloride (Farmalabor, Pozzillo, Italy) salt was used to formulate microcapsules as a crosslinker, which linked sodium alginate chains and formed a solid gel.

### 2.2. Extracts and Essential Oil Preparation

#### 2.2.1. Preparation of *Glycyrrhiza glabra* L. Extract

*Glycyrrhiza glabra* L. extract was prepared by using dried and milled plant root powder. The extraction was done using 1 g ± 0.001 g of powder in 10 mL of purified water. First, the plant material was macerated in water for 4 h. After that, ultrasound-assisted extraction was performed using an ultrasound bath (frequency 38 kHz) (Grant Instruments™, XUB12 Digital, Cambridge, England). The extraction time was 30 min; the processing temperature was 40 ± 2 °C. After extraction, samples were centrifuged for 10 min at 3382 g (5500 rpm) followed by the decantation of the supernatant. The extract was filtered through the paper filter and used in the following research.

#### 2.2.2. Preparation of *Trifolium pratense* L. Extract

Before use, clover flowers were ground to a fine powder using an Ultra Centrifugal Mill ZM 200 (Retsch, Haan, Germany). Grinding was performed at 4025 g (6000 rpm) using a 0.5 mm trapezoid hole sieve. The extraction was performed as in the previous study using excipient β-cyclodextrin (β-CD) [36].

Ultrasound-assisted extraction was performed using an ultrasound bath (38 kHz) (Grant Instruments™ XUB12 Digital, Cambridge, England). A sample of 0.3 ± 0.001 g of dried and milled flower heads was macerated in 10 mL of 50% ethanol. Additionally, 0.1 ± 0.001 g of β-CDs was added to the extraction mixture samples (10 mL) to prepare samples with CD concentrations of 1% (*w*/*v*). Ultrasound extraction time was 10 min, and the processing temperature was 40 ± 2 °C. After ultrasound processing, the samples were put in a round bottom flask and refluxed in a sand bath at 100 °C for 1 h. After that, the mixture was left to cool at a temperature of 25 ± 2 °C. The samples were centrifuged for 10 min at 3382 g (5500 rpm), followed by the decantation of the supernatant. The extract was filtered through the paper filter and used in the following research.

#### 2.2.3. Essential Oil Preparation

*Myristica fragrans* Houtt. essential oil was prepared by modified hydrodistillation. *Myristica fragrans* Houtt. seed powder was mixed with magnesium aluminometasilicate, and then distilled water was added to the mixture (5:1:300). Hydrodistillation with Clevenger-type apparatus (manufacturer of apparatus Winzer Corporation, bought from Carl Roth, Drogen, Germany) and heating mantle (Pilz^®^, Winkler, Germany) was carried out for 2 h. A colourless essential oil was obtained and collected in an airtight bottle. The essential oil was stored in a refrigerator at 4 °C until needed.

### 2.3. Chromatografical Analysis of Extracts

#### 2.3.1. HPLC–PDA Conditions

High-performance liquid chromatography with diode array detectors (HPLC-PDA) was performed as described in previous work by Kazlauskaite et al. [36]. It was carried out using the Shimadzu Nexera X2 LC-30AD HPLC system (Shimadzu, Tokyo, Japan), equipped with an SPD-M20A diode array detector (DAD).

For the determination of polyphenols in plant extracts, an ACE 5 C18 250 × 4.6 mm column (Advanced Chromatography Technologies, Aberdeen, Scotland) was used. The mobile phase consisted of solvent A (acetic acid/methanol/deionised water) (1:10:89 *v*/*v*/*v*) and solvent B (acetic acid/methanol) (1:99 *v*/*v*). The linear gradient elution profile was as follows: 80% A/20% B at 0 min, 30% A/70% B at 30 min, and 90% A/10% B at 39 to 40 min. The flow rate was 1 mL/min, and the injection volume was 10 μL. Absorption was measured at 260 nm. Quantification of compounds was performed using reference standards.

#### 2.3.2. GC-MS Conditions

Gas chromatography-mass spectrometry (GC-MS) analysis was performed as described in Matulyte et al. [37]. GC-MS was carried out using the GCMS-QP2010 system (Shimadzu, Tokyo, Japan). The column used in the process was RTX-5MS, 30 m × 0.25 mm i.d. ×0.25 µL film thickness. The flow rate of helium (99.999%, AGA Lithuania) carrier gas was set at 1.23 mL/min.

A total of 20 μL of the sample (extract or essential oil) was diluted to 1 mL with hexane (≥99%, Sigma-Aldrich, Germany). The oven temperature was maintained at 40 °C for 2 min after injection and then programmed at 3 °C/min to 210 °C, at which the column was maintained for 10 min. The split ratio was 1:10. The mass detector electron ionisation was 70 eV. Identification of volatile compounds was carried out using mass spectra library search (NIST 14).

### 2.4. Total Phenolic and Flavonoid Content and Antioxidant Activity

Total phenolic and flavonoid content and antioxidant activity methods ABTS, DPPH, and FRAP were carried out as described in Kazlauskaite et al. [36].

#### 2.4.1. Determination of Total Phenolic Content

A total of 0.5 mL of extract was mixed with 2.5 mL of 1:9 diluted Folin–Ciocalteu’s phenol reagent and 2.0 mL of 7% (*w*/*v*) sodium carbonate. Absorbance was measured at 765 nm after 1 h using a spectrophotometer (Shimadzu UV-1800, Kyoto, Japan). The calibration curve was prepared using gallic acid (0–0.1 mg/g; y = 11.108; R^2^ = 0.9981). The results were expressed as gallic acid equivalent per gram dry weight (mg GA/g dw).

#### 2.4.2. Determination of Total Flavonoid Content

A total of 0.1 mL of extract was added to 1.0 mL 96% (*v*/*v*) of ethanol, 0.05 mL of 33% acetic acid, 0.15 mL 10% aluminium chloride, and 2.0 mL 5% hexamethylenetetramine solutions. Spectrophotometric analysis was performed after 30 min at 475 nm wavelength using a spectrophotometer (Shimadzu UV-1800, Kyoto, Japan). The results were expressed as rutin equivalent per gram dry weight (RE/g dw) and calculated by the formula TFC = C · V_e_ · F/M, where TFC is the total flavonoid content (mg RE/g dw); C is the concentration of the used standard (mg/L); V_e_ is the volume of the used solvent (L); F is the dilution coefficient of the sample; and M is the mass of the sample (g). The calibration curve was obtained with a rutin (0–0.5 mg/g; y = 5.0867; R^2^ = 0.9985). The results were expressed as rutin equivalent per gram dry weight (RU/g dw).

#### 2.4.3. ABTS Radical Scavenging Activity Assay

Aqueous ABTS solution (7 mM) was mixed with potassium persulfate (2.45 mM) solution and stored in the dark for 12–16 h to produce a dark-coloured solution containing ABTS radical cation. ABTS working solution was prepared using ABTS radical solution and diluting it with water. Working solution absorbance should be regulated to 0.90 (±0.02) at 734 nm using a spectrophotometer (Shimadzu UV-1800, Kyoto, Japan).

Free radical scavenging activity was evaluated by mixing 2.0 mL of ABTS working standard with 200 μL of the test sample in the cuvette. The samples were incubated in the dark at room temperature for 30 min. The calibration curve was obtained with a Trolox (0–0.5 mg/g; y = 0.0001728x; R^2^ = 0.9832). The results were expressed as Trolox equivalent per gram dry weight (TE/g dw).

#### 2.4.4. DPPH Radical Scavenging Activity Assay

A total of 2.0 mL of DPPH solution (0.1 mM in ethanol) was mixed with 2.0 mL of the samples. The reaction mixture was shaken and incubated in the dark at room temperature for 30 min, and the absorbance was read at 517 nm against the blank using a spectrophotometer (Shimadzu UV-1800, Kyoto, Japan). The calibration curve was obtained with a Trolox (0–0.016 mg/g; y = 0.00623x; R^2^ = 0.9923). The results were expressed as Trolox equivalent per gram dry weight (TE/g dw).

#### 2.4.5. Ferric Reducing Antioxidant Power (FRAP)

The FRAP assay was prepared by mixing 0.3 M acetate buffer, 10 mM TPTZ solution with 40 mM HCl, and 20 mM ferric chloride solution. A total of 10 μL of the sample was combined with 200 μL of FRAP reagent; the contents were mixed vigorously. The absorption was measured at 593 nm using a spectrophotometer (Shimadzu UV-1800, Kyoto, Japan). The calibration curve was obtained with ferrous sulfate (0–1 mg/g; y = 2.6272; R^2^ = 0.9985). The results were expressed as ferrous sulfate equivalent per gram dry weight (FS/g dw).

### 2.5. Antimicrobial Activity

Antimicrobial activity was determined with the diffusion method in a solid nutrient media of agar. Mueller–Hinton Agar (Mueller–Hinton II Agar, BBL, Cockeysville, MD, USA) was used.

Standard cultures of nonspore bacteria (all bacteria were obtained from American Type Culture Collection (ATCC))—*Staphylococcus aureus* (ATCC 25923; human nasal microbiota), *Staphylococcus epidermidis* (ATCC 12228; human skin microbiota), *Enterococcus faecalis* (ATCC 29212; human colonic microbiota), *Escherichia coli* (ATCC 25922; human colonic microbiota), *Klebsiella pneumoniae* (ATCC 13883; human microbiota), *Pseudomonas aeruginosa* (ATCC 27853; human microbiota), and *Proteus vulgaris* (ATCC8427; human microbiota). Bacteria were grown for 20–24 h at 35–37 °C on Mueller–Hinton Agar. The bacterial suspension was prepared from cultures of cultivated bacteria in sterile physiological sodium chloride (0.9%) solution, standardised with a McFarland standard indicator. The bacterial suspension was considered standardised when the indicator value was 0.5 (1 mL of bacterial suspension contains 1.5 × 10^8^ cells of the micro-organism).

Standard spore bacteria cultures of *Bacillus cereus* (ATCC 6633; soil microbiota) were grown for 7 days at 35–37 °C on Mueller–Hinton Agar. After growing the culture of spore bacteria, it was washed off the surface of the medium with a sterile physiologic solution. The prepared suspension was heated for 30 min at 70 °C and diluted with physiological saline until the spore concentration in 1 mL was between 10 × 10^6^ and 100 × 10^6^.

The standard culture of the fungus *Candida albicans* (ATCC 10231; human microbiota) was grown for 20 to 24 h at 30 °C for 72 h on Sabouraud agar. The fungal suspension was prepared from cultivated fungal cultures in physiological saline and standardised with a McFarland standard indicator.

A 0.5 McFarland turbidity suspension of the standard bacteria was prepared. The bottom of the Petri dishes was divided into 9 segments. The technology of reference microorganisms to Mueller–Hinton agar was used to determine the antimicrobial activity of *Glycyrrhiza glabra* L. and *Trifolium pratense* L. extracts. The disk method was used to determine the antimicrobial activity of *Myristica fragrans* Houtt. essential oil.

#### 2.5.1. Antibacterial Activity of Extracts

The chosen amount of extract was poured into a sterile Petri dish (*Glycyrrhiza glabra* L. extract from 1 mL to 0.0075 mL; clover from 1 mL to 0.1 mL). Then, 5 mL of Mueller–Hinton agar was added. After the solidification of the agar, suspensions of reference microorganisms were inoculated. Samples were kept in a thermostat for 20–24 h at 35 °C, then stored for 24 h at room temperature. Antimicrobial activity was evaluated. If the cultures had grown—the sample did not inhibit the growth of bacteria. If the reference culture did not grow, the sample has an antimicrobial effect against the microorganism.

#### 2.5.2. Antibacterial Activity of Essential Oil

A total of 0.5 mL of bacterial suspensions were separately poured into sterile Petri dishes. After that, 5 mL of Mueller–Hinton agar was poured into the plates. After the agar solidified, an 8 mm disk was placed on top, and 30 µm of *Myristica fragrans* Houtt. essential oil was added. Samples were kept in a thermostat for 20–24 h at 35 °C, then stored for 24 h at room temperature. Antimicrobial activity was then evaluated: if the cultures grew around and under the disk—the sample did not inhibit the growth of bacteria. If the reference culture did not grow, the sample has an antimicrobial effect against the microorganism.

### 2.6. Antiviral Activity

Dr. I. Jacevičienė provided a Vero cell line (ATCC CCL-81) from the Department of Virus Research at the National Food and Veterinary Risk Assessment Institute in Lithuania. The cells were cultivated in Dulbecco’s modified Eagle’s medium (DMEM, Gibco, TX, USA) supplemented with 10% foetal bovine serum (FBS, Gibco, TX, USA) at 37 °C in a 5% CO_2_ incubator. Gentamycin (50 μg/mL, Gibco, TX, USA) and nystatin (100 units/mL, Gibco, TX, USA) were used to prevent microbial contamination.

The Vero-adapted Beaudette infectious bronchitis virus (IBV) strain was used. Dr. M.H. Verheije of Utrecht University in The Netherlands provided the virus. The virus stocks were prepared and stored at −80 °C in aliquots.

Determination of TCID50 of the control and the treated IBV were performed in 96-well plates (TPP, Switzerland) with Vero cells. Serial dilutions of IBV in tenfold steps were prepared. Each sample was tested in octuplicates, and the experiments were repeated twice. CPE was evaluated after 72 h. Virus titres and standard deviations were calculated using the Kärber method (Kärber 1931), and the virus reduction capacity was assessed [38].

The cytotoxicity assay was performed to determine the 50% cytotoxic concentration (CC_50_). CC_50_ was determined for each extract on Vero cells using an MTT assay [39]. First, cells were seeded at a concentration of 1 × 10^4^ cells/well in a 96-well plate and grown at 37 °C for 1 day. The assay was performed in octuplicate for each extract. After 72 h, the MTT reagent (10 μL, 5 mg/mL, Sigma-Aldrich, St. Louis, MO, USA) was added and incubated for 4 h at 37 °C. A total of 100 µL dimethyl sulfoxide was added to each well, and the plates were placed on the shaker for 5 min. The absorbance of each well was measured at 620 nm in a microplate reader (Thermo Scientific™, Multiskan™ FC Microplate Photometer, Tokyo, Japan), and the percentage of cell survival was calculated. The relative viability in untreated cells was set as 100%. Finally, dose–response curves were plotted to calculate CC_50_ that caused lysis and death of 50% of cells (Chang, 2016). The dilutions of plant extracts were prepared for screening and determination of antiviral and virucidal activity.

Testing solutions were prepared by mixing IBV (10^5.25^ TCID_50/mL_) suspensions in DMEM with previously established noncytotoxic extract volumes (dilutions 1:15 and 1:30). The controls of diluents of plant preparations were used. The virus and extract mixtures were incubated at 20 °C for 1 h and then titrated as described above. Controls of cells, the virus, and extracts were included. After 72 h of incubation, the plates were examined using an inverted microscope (Leica, Germany) to detect CPE. The reduction factor was used to describe plant preparations’ viral reduction potential [38]. The reduction factor ≤ 1 log_10_—regarded as insignificant; 1–2 log_10_—indicative/contributable; 2–4 log_10_—moderate; and >4 log_10_—high.

Antiviral activity was evaluated by infection of cells after virus treatment, during virus treatment, and the treatment of cells before infection and after infection. A total of 0.05 IBV particles per cell multiplicity of infection (MOI) was used. Prepared extracts were serially diluted twofold per seven wells in DMEM and assessed for their ability to inhibit IBV replication using four mechanisms.

In the first method, IBV was treated with the diluted extract for 1 h in a separate 96-well plate and then poured onto the cells. After incubating for 1 h at 37 °C in 5% CO_2_, the mixtures were discarded, and then the cells were washed twice with PBS. After washing, 200 µL of DMEM containing 2% FBS was added. Observation by microscopy for inhibition of CPE was performed after incubation for 72 h at 37 °C in 5% CO_2_.

In the second method, the diluted extract and IBV mixtures were poured onto the cells immediately. After incubating for 1 h at 37 °C in 5% CO_2_, the mixtures were discarded, and then the cells were washed twice with PBS. After washing, 200 µL of DMEM containing 2% FBS was added. Observation by microscopy for inhibition of CPE was performed after incubation for 72 h at 37 °C in 5% CO_2_.

In the third method, the cells were inoculated with the IBV and then treated with the extract. First, the cells were inoculated with the virus and incubated for 1 h at 37 °C in 5% CO_2_. Then the unadsorbed IBV was discarded, and the cells were washed twice with PBS. After washing, the cells were treated with the diluted extracts for 1 h at 37 °C in 5% CO_2_. After washing the cells twice with PBS, 200 µL of DMEM containing 2% FBS was added. Observation by microscopy for inhibition of CPE was performed after incubation for 72 h at 37 °C in 5% CO_2_.

In the fourth method, the cells were treated with extract before inoculation. First, the cells were treated with the diluted extracts for 1 h at 37 °C in 5% CO_2_. Then the cells were washed twice with PBS and inoculated with IBV. After incubation for 1 h at 37 °C in 5% CO_2_, the cells were washed twice with PBS, and 200 µL of DMEM containing 2% FBS was added. Observation by microscopy for inhibition of CPE was performed after incubation for 72 h at 37 °C in 5% CO_2_.

Every sample of the extract was tested twice in quadruplicate.

The cytopathic effect (CPE) of IBV was evaluated by optical microscopy. The endpoint was the extract dilution that inhibited 100% of the CPE at two noncytotoxic concentrations.

### 2.7. Emulsion Preparation

First, a 4% sodium alginate solution was prepared from distilled water and alginic acid sodium salt. It was used throughout the experiment for emulsion preparation as the shell material. Emulsion with *Trifolium pratense* L. and *Glycyrrhiza glabra* L. extracts, and *Myristica fragrans* Houtt. essential oil were prepared as follows: solution with excipients (maltodextrin, inulin, and/ or gum Arabic) was mixed with sodium alginate solution (stirred for 15 min with a magnetic stirrer MSH-20A (Witeg, Wertheim, Germany)) and then extracts with essential oil were added. The solution was homogenised for 15 min at 5000 rpm using an IKA T18 homogeniser (IKA-Werke GmbH & Co., KG, Staufen, Germany).

The emulsion’s stability was tested using a centrifuge Sigma 3-18KS (Sigma Laborzentrifugen GmbH, Osterode am Harz, Germany). The test was repeated three times using 23 °C temperature, 3000 rpm, and the duration was 5 min. The centrifugation index (CI) was calculated to evaluate emulsion stability.

(1) $$\mathrm{CI}\left(\%\right)=\frac{V_{e}}{V_{i}}\cdot 100$$

where V_e_ is the volume of the remaining emulsion after centrifugation and V_i_ is the volume of the initial emulsion.

### 2.8. Particle Size and Distribution Measurements

*Myristica fragrans* Houtt. essential oil particles’ size and distribution were assessed using Mastersizer 3000 with a Hydro EV unit (Malvern analytical Ltd., Malvern, UK). The emulsion was added dropwise in the dispersant (water) to obtain laser obscuration between 9.5% and 10.5%. The pump speed was kept constant at 2400 rpm. The refractive index used for dispersant and dispersing material was 1.330 and 1.478, respectively. The average was calculated for particle size distribution in five runs. The percentile (D10, D50, and D90) values described the formulations.

### 2.9. Microcapsule Preparation Using the Extrusion Method

Microcapsules were prepared by extrusion method. The medical syringe (Jiangsu Zhengkang Medical Apparatus, Yangzhou, China) and NE-1000 Programmable Single Syringe Pump (KF T technology SRL, Rozzano, Italy) were used to prepare droplets for microcapsules. The drops of emulsion were ejected from the needle into the crosslinker solution. The height from the needle to the solution surface was 10–15 cm, and the pumping speed was 3 mL/min. A solution of 5% calcium chloride was used as a crosslinker. Microcapsules were prepared by using a magnetic stirrer. Particles in the crosslinker solution were stirred for 15 min, and then the microcapsules were filtered using filter paper and washed with distilled water. Manufactured capsules were left to dry at room temperature (20 ± 2 °C) for 24 h. Dried and wet microcapsules were stored in sealed tubes until further tests.

### 2.10. Physical Parameters of Microcapsules

#### 2.10.1. Size of Dry and Wet Microcapsules

The microcapsules’ size was measured using a Digital Calipiper micrometre (BGS technic, Wermelskirchen, Germany). The diameter of 30 units of dried and freshly made capsules was measured, and the average was calculated.

#### 2.10.2. Firmness of Microcapsules

The force of firmness was measured on freshly made microcapsules by texture analyser TA.TX.plus (Texture Technologies, Hamilton, MA, USA). For one sample, 5 units of microcapsules were taken, and the force which was required to compress 2 mm was measured using a P/100 probe.

#### 2.10.3. Swelling Characteristic of Microcapsules

Dried microcapsules were swelled in gastric and intestinal media [33]. The microcapsules were weighed at successive time intervals of 0, 0.5, 1, 2, 4, and 24 h. The swollen microcapsules were removed and filtered using metal mesh and dried with a paper towel to eliminate the excess fluid. To determine the swelling index (SI), the following formula for calculation was used:

(2) $$\mathrm{SI}\left(\%\right)=\frac{W_{s}-W_{i}}{W_{i}}\cdot 100$$

where W_s_ is the weight of swollen microcapsules at the time and W_i_ is the dried microcapsules’ weight.

### 2.11. Statistical Data Analysis

Data were analysed using SSPS version 20.0 (IBM Corporation, Armonk, NY, USA). All experiments were performed in triplicate. Data are expressed as mean ± standard deviation (SD). The comparisons between three different measurements were made using Friedman and Wilcoxon tests. The results were considered statistically significant at *p* < 0.05. For antiviral experiments, the differences between the methods and extracts were evaluated by Fisher’s criteria and the Student’s t-test. The data were regarded as significant when *p* < 0.05.

## 3. Results and Discussion

### 3.1. Quantification of Main Compounds in Plant Extracts and Essential Oil

The main compound with higher biological activity in *Glycyrrhiza glabra* L. extract is glycyrrhizic acid [40]. *Glycyrrhiza glabra* L. extract was made by maceration combined with ultrasound processing. The extracts yielded 349.29 ± 14.55 µg/g glycyrrhizic acid. The extract was concentrated by evaporation of water (half the weight of the extract), and the glycyrrhizin content determined after concentration was 2.07-fold higher. In the literature, there are other extraction methods to obtain glycyrrhizin. Using sonification and pressurised hot water extraction, the amount of glycyrrhizin acid was 17.21 ± 1.27 mg/g and 13.20 ± 2.37 mg/g, respectively [41]. A total of 36.4 µg/g of glycyrrhizic acid was obtained by ultrasound-assisted extraction (30:1 solvent to solute ratio, 40 °C temperature) [42]. Ultrasound alone yields lower concentrations of glycyrrhizic acid; therefore, combining it with maceration gives better results.

*Trifolium pratense* L. flower extract (extraction with excipient *β*-CD) contained 171.57 ± 12.36 µg/g of genistein and 393.23 ± 23.71 µg/g of daidzein. This amount of isoflavones is higher than the average reported in the literature. It was reported that petioles contained 0.028 mg/g of daidzein and 0.054 mg/g of genistein, and flowers contained 0.44 µg/g of genistein and daidzein, except for minimal traces were not detected [43]. Using reflux (85 °C) extraction, 0.11 ± 00.28 mg/g of daidzein and 0.10 ± 0.0053 mg/g of genistein were obtained (extrahent was methanol). These concentrations were a lot lower than those obtained in this study. Adding excipient *β*-CD to extraction media helps extract target compounds. β-CDs form host–guest inclusion complexes with various drugs in a solution or a solid state. The existence of a β-CD hydrophobic cavity boosts the extraction of phenolic compounds, including isoflavones [44]. Clover extract was also concentrated by evaporating water (50%), and the isoflavones concentration found in the sample were genistein 265.46 µg/g and daidzein 598.16 µg/g.

*Myristica fragrans* Houtt. essential oil (prepared by modified hydrodistillation; origin country: Grenada) had the highest concentration of sabinene 42.55%, α-pinene 13.58%, β-terpinene 8.17%, and β-myrcene 3.47%. The essential oil yield was 10.88 ± 0.75%. Compared with other studies, it was found that *Myristica fragrans* Houtt. essential oil yield prepared using hydrodistillation was from 3.2% to 10.3% and when prepared by steam distillation was 0.3–12.5% [10]. The essential oil isolated from the seeds of *Myristica fragrans* Houtt. (origin country Nigeria) was found to contain 49.09% sabinene, 13.19% α-pinene, 6.72% α-phellandrene, and 6.43% terpinen-4-ol as major constituents [45]. Therefore, the monoterpenes identified from *Myristica fragrans* Houtt. essential oil in our sample contained higher concentrations. *Myristica fragrans* Houtt. Essential oil from Brasilia had an even lower amount of sabinene (25.0%) but a higher amount of myrcene (10.9%) compared with this study’s results [46].

### 3.2. Phenols and Antioxidant Activity of Extracts and Essential Oil

Many components in extracts and essential oil, i.e., carotenoids, vitamin C, vitamin E, and phenolic compounds, contribute to the overall antioxidant activity; it is difficult to measure the total antioxidant activity based on individual active components. This is because compounds can interact with each other to produce antagonistic or synergistic effects [47]. As a result, the extracts were tested for total phenolic compounds, flavonoid content, and antioxidant activity using three different methods—ABTS, DPPH, and FRAP. The research was done using concentrated *Glycyrrhiza glabra* L. and *Trifolium pratense* L. extracts.

#### 3.2.1. Total Phenolic and Flavonoid Content

The total phenolic content found in *Glycyrrhiza glabra* L. extract was 43.14 ± 0.06 mg GA/g dw. Higher results were obtained from the *Trifolium pratense* L. flowers extract—74.00 ± 0.15 mg GA/g dw (Table 1). Ethanolic *Trifolium pratense* L. extract had a significantly higher total phenol content compared to aqueous *Glycyrrhiza glabra* L. extract (*p* < 0.05), and the exact correlation was observed comparing total flavonoids. Varying solubility of the phenolic compounds due to different solvents could be explained by the solvent polarity, and ethanol is more efficient in extracting lower molecular weight polyphenols, especially glycosides [48,49].

The essential oil of *Myristica fragrans* Houtt., because of its preparation method, contained only a small amount of phenols (7.49 ± 0.04 mg GA/g dw). The main compounds found in our sample were monoterpenes (identified as 67.77%). This correlates with the results published by other researchers [10,15]. The total flavonoids determined in the essential oil was 6.84 ± 0.05 mg RU/g dw.

#### 3.2.2. Antioxidant Activity of Extracts and Essential Oil

In the literature, a few antioxidant assays have been developed based on methodological differences to detect antioxidant activity in samples taken from natural sources, including extracts and essential oils. In this study, DPPH, ABTS, and FRAP methods were used to determine the antioxidant potentials of *Glycyrrhiza glabra* L. and *Trifolium pratense* L. extract, also *Myristica fragrans* Houtt. essential oil. Radical scavenging antioxidants are necessary for the antioxidative defence to protect cells from the injurious effects of free radicals [50]. Antioxidants can delay the progress of many chronic diseases as well as lipid peroxidation [51].

The antioxidant activity of *Glycyrrhiza glabra* L. and *Trifolium pratense* L., using the DPPH method, was similar (Table 2). *Trifolium pratense* L. extract showed better antioxidant activity using the ABTS method (638.55 ± 9.14 µg TE/g dw), but *Glycyrrhiza glabra* L. extract had higher ferric reducing power (675.71 ± 4.61 mg FS/g dw). Studies in the literature have shown that both *Trifolium pratense* L. and *Glycyrrhiza glabra* L. are good antioxidants. *Trifolium pratense* L. is known to contain isoflavones (daidzein, genistein, formononetin, and biochanin A), which possess estrogenic effects, but antioxidant properties of extract can be related to quercetin, hyperoside, clovamides, and other phenolic compounds [52]. The reported main phenolic compounds in *Glycyrrhiza glabra* L. extract responsible for its antioxidant activity are also isoflavones, such as glabridin, hispaglabridin A, and 30-hydroxy-4-O-methylglabridin [53].

*Myristica fragrans* Houtt. essential oil reported possessing antioxidant activity as well as *Trifolium pratense* L. or *Glycyrrhiza glabra* L. extracts. Nevertheless, the concentration of the essential oil used in this study was 1%. The isomers α-pinene and β-pinene possess antioxidant and antimicrobial activity, but the antimicrobial activity of β-pinene is more robust [17].

### 3.3. Antimicrobial and Antiviral Activity

The antimicrobial activity of *Trifolium pratense* L. and *Glycyrrhiza glabra* L. extract was determined using nonconcentrated extracts. *Glycyrrhiza glabra* L. extract had expressed antimicrobial activity against Gram-positive bacteria: *Staphylococcus aureus*, *Staphylococcus epidermidis*, *Enterococcus faecalis*, and *Bacillus cereus*. Additionally, it suppressed the growth of the fungus *Candida albicans*.

A concentration of 0.2% extract inhibited these pathogens, whereas *Bacillus aureus* was inhibited by a 2% *Glycyrrhiza glabra* L. extract solution. *Glycyrrhiza glabra* L. extract had no antimicrobial activity against Gram-negative bacteria: *Escherichia coli*, *Klebsiella pneumoniae*, *Pseudomonas aeruginosa*, and *Proteus vulgaris*. The results are presented in Table 3.

The antimicrobial activity of *Trifolium pratense* L. extract was not expressed, but the obtained results showed that this extract inhibits gram-positive bacteria more. 0.75 mL of extract in 5 mL of agar medium (13.04% concentration of extract) inhibited the growth of *Staphylococcus epidermidis* and inhibited *Staphylococcus aureus* and *Bacillus cereus* weakly. The growth of other pathogens was not inhibited (Table 4).

According to the literature, the extraction solvent capability to extract active substances of the active principles has a major importance on the antibacterial and antifungal activity of the *Trifolium* species. Studies on the antimicrobial properties of *Trifolium pratense* L. included a comparison of the actions of different extracts solvents, concentration of the extract and drying conditions [19,54,55,56]. In our study case, the concentration of isoflavones, which are responsible for antibacterial effect in *Trifolium pratense* L. was too low to have strong antibacterial response.

Essential oils are volatile liquids, so it is difficult to determine their effect due to evaporation. One essential oil concentration was chosen in this study, and antimicrobial activity against 9 pathogens was evaluated. Results (Table 4) showed that Myristica fragrans Houtt. Essential oil suppressed the growth of half of referenced gram-positive and half of referenced gram-negative pathogens (*Staphylococcus aureus*, *Bacillus cereus* and *Escherichia coli*, *Klebsiella pneumoniae,* respectively), and the other pathogens were not suppressed (two of gram-positive and two of gram-negative).

Observing the results obtained by other scientists and comparing it with this study, it was found that a two times higher amount of nutmeg essential oil suppressed *Candida albicans*, *Staphylococcus aureus*, *Bacillus cereus*, *Bacillus luteus*, *Escherichia coli*, and *Pseudomonas aeruginosa* [17]. In the other study, it was found that *Myristica fragrans* Houtt. essential oil was more effective in inhibiting the growth of *S. aureus* than gentamicin (sample and gentamicin (as control) concentrations were the same, and the zone of inhibition of *Myristica fragrans* Houtt. was 1.22% greater than the antibiotic) [57].

In the literature, it was reported that *Trifolium pratense* L. extract suppressed *Bacillus subtilis* growth. The diameter of the nongrowth zone was 20 mm, while the essential oil nongrowth zone was 12 mm. The extract had 40% higher activity against *B. subtilis* compared to essential oil [58]. The antibacterial activity of the essential oil against *Staphylococcus aureus*, *Escherichia coli*, *Salmonella typhimurium*, and *Bacillus cereus* was also tested, and it was found that clover extract did not inhibit the growth of either pathogen [59]. Thus, it can be concluded that to increase the antibacterial activity in products, clover extract should be used instead of essential oil.

In publications about *Glycyrrhiza glabra* L. roots, it was revealed that an ethanolic extract of *Glycyrrhiza glabra* L. had a positive effect on suppressing *B. cereus*, *B, subtilis*, *K. pneumoniae*, and *S. aureus*; however, the ethanolic extract did not suppress *E. faecalis* growth [60]. In our study it was found that 0.01 mL of the *Glycyrrhiza glabra* L. extract in a 5 mL agar medium effectively suppressed *E. faecalis*.

### 3.4. Antiviral Activity of Used Plant Extracts and Essential Oil

It was determined that the CC_50_ of *Trifolium pratense* L. was 4.8 mg/mL and the CC_50_ *Glycyrrhiza glabra* L. was 10.0 mg/mL for Vero cells. The diluent of *Glycyrrhiza glabra* L. had no virucidal effect upon IBV, whereas the diluent, ethanol, used for *Trifolium pratense* L. at concentrations of 8.0% ethanol (*Trifolium pratense* L. with 4.8 mg/mL) and 6.67% ethanol (*Trifolium pratense* L. with 4.0 mg/mL) had a virucidal effect (Table 5). The diluent of the almond EO did have a positive impact on virucidal activity (*p* < 0.05).

Based upon cytotoxicity and control results, dilutions of 1:15 and 1:30 were chosen for comparison of the virucidal activity of extracts.

All plant preparations (1:15 and 1:30) showed a ≥90% reduction of the viral titre and had moderate or contributable virucidal activity. At 1:15 dilution (2 mg/mL), *Trifolium pratense* L. showed moderate virucidal activity (virus reduction 2.75 log_10_), while at a 1:30 dilution (1 mg/mL), virus reductions were between 1.125 log_10_ and 1.75 log_10_. Other plant preparations showed contributable virucidal activity at both dilutions. The virucidal activity of *Trifolium pratense* L. and *Glycyrrhiza glabra* L. was dose-dependent (*p* < 0.05), while the concentration of almond had no effect (*p* > 0.05).

Antiviral activity was tested for the extracts of *Trifolium pratense* L. and *Glycyrrhiza glabra* L. (Table 6). It was shown that the extracts were effective against IBV and could protect the Vero cells prior to or during infection. IBV pretreatment with extracts after infection and cell pretreatment prior to infection could not inhibit 100% of the CPE. Combining these extracts together can result in a promising preparation that will have high antioxidant, antibacterial, and antiviral properties.

### 3.5. Emulsion Physical Stability

**Figure 1:**
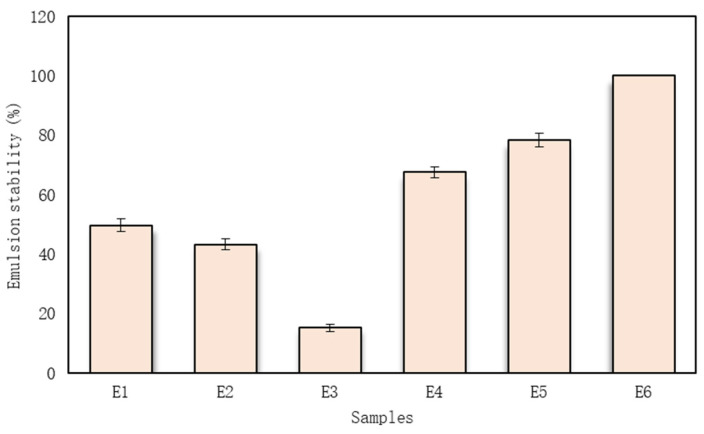
The emulsion stability (centrifugation index). The code of emulsion is given in Table 7.

Six emulsion samples were prepared, and their stability was evaluated (the emulsion’s composition is shown in Table 7). Sample E3 became an inhomogeneous texture after all ingredients were mixed, and its centrifugation index was the lowest (Figure 1).

When comparing the stability of the emulsion, it was established that one excipient (maltodextrin/inulin/gum Arabic) was unable to keep the emulsions stable (CI was lower than 50%). Then, two different excipients were added to the composition, and the stability of the emulsion was increased by approximately 46% compared with E1. The highest stability of the emulsion was evaluated, and then three excipients were added. Emulsion E6 was the most stable, and its CI was 100%.

**Figure 2:**
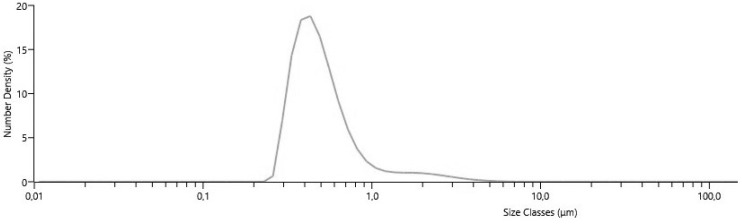
Particle size distribution.

In an emulsion, particle size, distribution, and essential oil drop diameter were determined using the Mastersizer 3000 (Figure 2). *Myristica fragrans* Houtt. essential oil drop diameter in the emulsion was 0.571 µm. Evaluated percentiles were measured: D10 was 0.328 ± 0.051 µm, D50 was 0.46 ± 0.03 µm, and D90 was 0.89 ± 0.094 µm. The percentiles D10, D50, and D90, indicate the size for which 10%, 50%, and 90% of the particles are equal to or less, respectively [61]. The emulsion particle size with different essential oils was 16.70–55.56 µm. The base was made from soy protein isolate and gum Arabic [62]. Studies similar to this, with *Myristica fragrans* Houtt. essential oil, extracts, and gum Arabic, maltodextrin, and inulin, were not found. This stable emulsion (E6) was used to prepare microcapsules by extrusion.

### 3.6. Microcapsules’ Physical Parameters

**Figure 3:**
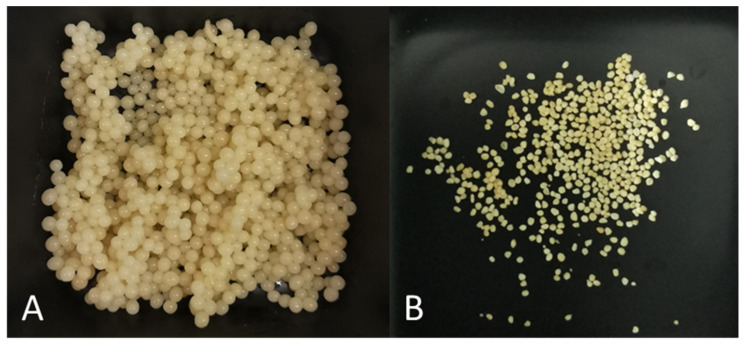
Extruded microcapsules (**A**) immediately after extrusion and (**B**) after drying the microcapsules.

In a 5% crosslinker solution, microcapsules were formulated (Figure 3), and all microcapsules were spherical and of light brown colour. The yield of microcapsules was 68.67 ± 3.08%. The freshly prepared microcapsules’ diameter was 1.87 ± 0.35 mm and soft (easily crushed between the fingers). The force of crushing (measured with a Texture analyser) was 3758.52–4135.87 g. The dried microcapsules’ diameter was 0.76 ± 0.11 mm, and the device did not measure the force because the maximum force value is 6500 g; dried microcapsules were harder than this value.

Extrusion technology to produce microcapsules with essential oils and extracts is rarely used, but similar research results have been found. In a study with *Myristica fragrans* Houtt. essential oil, when polysorbate 80 is used as an emulsifier, microcapsules of size 2.120–2.280 mm with a strength of 4333.46–5116.70 g were formed in a 2% crosslinker solution [33]. Microcapsules with 1% of rosemary essential oil and without other excipients had a diameter between 0.950 mm and 0.756 mm (non-dry and dry, respectively; only sodium alginate 4% solution was used) [25].

**Figure 4:**
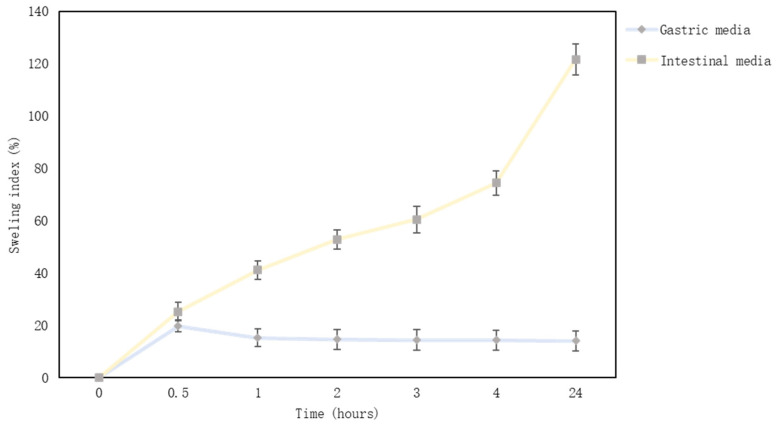
Microcapsules swelling graph in simulated intestinal and gastric media.

Microcapsules’ swelling index in gastric media was from 19.74% to 13.96%, and alginate microcapsules did not swell in gastric fluid. At first, the microcapsules swelled a little, but after 30 min, the swelling index decreased. Conversely, microcapsules swelled from 25.22% to 121.59% in the intestinal medium (Figure 4). After a day, the microcapsules had lost their shape and softened. Another study found that alginate microcapsules lost weight in the gastric media (alginate, soy protein microcapsules with thyme essential oil) [26]. The maximum swelling index was reached in 40–60 min, and then the amount of microcapsules in the gastric medium decreased [63]. Another study confirmed that alginate microcapsules swell in the intestinal medium, and the swelling index could be higher than 20 times [33]. In contrast, microcapsules almost did not swell, or their amount decreased in gastric media (pH = 1–2.5) [64]. The results of other studies are in agreement with the results obtained in this study and show that sodium alginate microcapsules swell in the intestinal medium. Sodium alginate is a polymer and forms hydrogels. It is well known that a hydrogel can respond to surrounding conditions such as pH, ionic strength, temperature, and electric current. The pH sensitivity is an important factor for controlled drug release in the gastrointestinal tract, which has a variation of pH from the stomach to the intestine. Many studies support the results that sodium alginate microcapsules or gels do not swell in the intestinal medium. The pH controls the degree of dissociation of the guluronic and mannuronic acid groups (alginate molecule parts); therefore, alginate does not swell in an acidic medium [65,66]. Alginate microcapsules can be used to develop gastric insoluble pharmaceutical formulations.

## 4. Conclusions

This study determined that the chosen plant extracts and essential oil had a significant concentration of active substances. The main component in liquorice extract was glycyrrhizin acid (349.29 ± 14.55 µg/g); the red clover extract contained 171.57 ± 12.36 µg/g of genistein and 393.23 ± 23.71 µg/g of daidzein. Nutmeg essential oil had four main chemical compounds: sabinene, α-pinene, β-terpinene, and β-myrcene.

All the plant extracts possess antioxidant effects. The highest effect was determined in the red clover extract, which also was the most effective against viruses. The highest antibacterial effect was found in the liquorice extract, which was most effective against Gram-positive pathogens. Therefore, plant-derived products can be considered promising when being used as antimicrobial agents. Extruded microcapsules with essential oil and extracts could be an additional form to incorporate in other pharmaceutical forms for therapeutic benefits. Considering the obtained research results, it is planned to evaluate the release of active compounds from microcapsules in the future.

## Figures and Tables

**Table 1:** The results of samples total phenol and flavonoid content.

Samples	Total Phenols (mg GA/g dw)	Total Flavonoids (mg RU/g dw)
*Glycyrrhiza glabra* L. extract	43.14 ± 0.06	16.89 ± 0.02
*Trifolium pratense* L. extract	74.00 ± 0.15	19.50 ± 0.04
*Myristica fragrans* Houtt. Essential oil (1%)	7.49 ± 0.04	6.84 ± 0.05

**Table 2:** Antioxidant capacity of plant extracts and essential oil.

Samples	DPPH (µg TE/g dw)	ABTS (µg TE/g dw)	FRAP (mg FS/g dw)
*Glycyrrhiza glabra* L. extract	26.22 ± 0.27	524.67 ± 6.32	675.71 ± 4.61
*Trifolium pratense* L. extract	26.27 ± 0.31	638.55 ± 9.14	526.86 ± 3.21
*Myristica fragrans* Houtt. Essential oil (1%)	8.69 ± 0.01	92.14 ± 1.26	176.05 ± 0.12

**Table 3:** Antimicrobial effect of *Glycyrrhiza glabra* L. extract on the growth of reference microorganism cultures.

Reference Cultures of Microorganisms	Amount of *Glycyrrhiza glabra* L. Extract (mL)									
	1.1	1.2	1.3	1.4	1.5	1.6	1.7	1.8	1.9	1.10
	1.0	0.75	0.5	0.25	0.1	0.075	0.05	0.025	0.01	0.0075
*Staphylococcus aureus* ATCC 25923	S	S	S	S	S	S	S	S	S	N
*Staphylococcus epidermidis* ATCC 12228	S	S	S	S	S	S	S	S	S	N
*Enterococcus faecalis* ATCC 29212	S	S	S	S	S	S	S	S	S	N
*Escherichia coli* ATCC 25922	N	N	N	N	N	N	N	N	N	N
*Klebsiella pneumoniae* ATCC 13883	N	N	N	N	N	N	N	N	N	N
*Pseudomonas aeruginosa* ATCC 27853	N	N	N	N	N	N	N	N	N	N
*Bacillus cereus* ATCC 11778	S	S	S	S	S	N	N	N	N	N
*Candida albicans* ATCC 10231	S	S	S	S	S	S	S	S	±	N
*Proteus vulgaris* ATCC 8427	N	N	N	N	N	N	N	N	N	N

S—inhibits the growth of reference culture of microorganisms (the antimicrobial effect was present); N—does not inhibit the growth of reference culture of microorganisms (no antimicrobial effect); ±—very weak growth.

**Table 4:** Antimicrobial effect of *Trifolium pratense* L. extract and *Myristica fragrans* Houtt. Essential oil on the growth of reference microorganism cultures.

Reference Cultures of Microorganisms	Amount of Clover Extract (mL)					Amount of *Myristica fragrans* Houtt. Essential Oil (µg)
	2.1	2.2	2.3	2.4	2.5	4
	1.0	0.75	0.5	0.25	0.1	30.0
*Staphylococcus aureus* ATCC 25923	S	±	N	N	N	S
*Staphylococcus epidermidis* ATCC 12228	S	S	N	N	N	N
*Enterococcus faecalis* ATCC 29212	N	N	N	N	N	N
*Escherichia coli* ATCC 25922	N	N	N	N	N	S
*Klebsiella pneumoniae* ATCC 13883	N	N	N	N	N	S
*Pseudomonas aeruginosa* ATCC 27853	N	N	N	N	N	N
*Bacillus cereus* ATCC 11778	S	±	N	N	N	N
*Candida albicans* ATCC 10231	±	N	N	N	N	S
*Proteus vulgaris* ATCC 8427	N	N	N	N	N	N

S—inhibits the growth of reference culture of microorganisms (the antimicrobial effect was present); N—does not inhibit the growth of reference culture of microorganisms (no antimicrobial effect); ±—very weak growth.

**Table 5:** Comparison of the virucidal effect of plant extracts on IBV.

Material, Dilution and Concentration			Virus Titre, TCID_50_	Virus Reduction (TCID_50_)		
				log_10_	%	Reduction Factor
Name	Dilution	Concentration				
*Trifolium pratense* L. extract	1:7.5	4 mg/mL	NA	NA	NA	NA
	1:15	2 mg/mL *	2.50 ± 0.26	2.72	99.82	moderate
	1:30	1 mg/mL *	3.50 ± 0.00	1.74	98.23	contributable
50% ethanol (control)	1:15	3.33%	5.22 ± 0.16	-	-	-
	1:30	1.67%	5.24 ± 0.16	-	-	-
*Glycyrrhiza glabra* L. extract	1:15	13.3 mg/mL *	3.50 ± 0.18	1.72	98.23	contributable
	1:30	6.6 mg/mL *	4.00 ± 0.19	1.24	94.38	contributable
Water (control)	1:15	6.66%	5.22 ± 0.16	-	-	-
	1:30	3.33%	5.24 ± 0.16	-	-	-
*Myristica fragrans* Houtt. essential oil **	1:15	13.3 µL/mL	4.13 ± 0.18	1.10	92.50	contributable
	1:30	6.6 µL/mL	4.00 ± 0.18	1.24	94.38	contributable
Almond oil (control)	1:15	13.3 µL/mL	3.88 ± 0.18	1.34	95.80	contributable
	1:30	6.6 µL/mL	3.75 ± 0.16	1.49	96.84	contributable
IBV control	undiluted	100%	5.25 ± 0.16	-	-	-

* Dose-dependent effect (*p* < 0.05). ** *Myristica fragrans* Houtt. essential oil was diluted in almond oil; the essential oil concentration was 1%. NA—nonapplicable because of ethanol virucidal effect.

**Table 6:** Antiviral activity of plant extracts.

Plant Extract	Antiviral Effect Evaluated by CIA100			
	Virus Pre-Treatment with Extract			Cell Pre-Treatment Prior to Infection
	Prior to Infection	During Infection	After Infection	
*Trifolium pratense* L.	+	+	-	-
*Glycyrrhiza glabra* L.	+	+	-	-

+: had an effect; -: no effect.

**Table 7:** Emulsions’ composition.

Ingredients	Samples and Ingredients Quantities					
	E1	E2	E3	E4	E5	E6
4% sodium alginate solution (g)	47.0	47.0	47.0	47.0	47.0	47.0
*Myristica fragrans* Houtt. essential oil (mL)	0.3	0.3	0.3	0.3	0.3	0.3
*Trifolium pratense* L. extract (mL)	4.2	4.2	4.2	4.2	4.2	4.2
*Glycyrrhiza glabra* L. extract (mL)	16.5	16.5	16.5	16.5	16.5	16.5
Maltodextrin (g)	12.0	-	-	8.6	10.3	6.2
Inulin (g)	-	12.0	-	3.4	-	4.3
Gum Arabic (g)	-	-	12.0	-	1.7	1.5
Purified water (mL)	20.0	20.0	20.0	20.0	20.0	20.0

## Data Availability

Data is contained within the article.
